# Draft genome sequence of multiple antibiotic-resistant *Vibrio harveyi* isolated from diseased olive flounder (*Paralichthys olivaceus*) farmed in South Korea

**DOI:** 10.1128/mra.00886-23

**Published:** 2024-01-08

**Authors:** Yu-Ra Kang, Nameun Kim, Yoonhang Lee, Jiyeon Park, Hyo-Young Kang, Ju-yeop Lee, Soo Ji Woo, Ahran Kim, Soo-Jin Kim, Myoung Sug Kim, Miyoung Cho, Do-Hyung Kim

**Affiliations:** 1 Department of Aquatic Life Medicine, Pukyong National University, Busan, South Korea; 2 Aquaculture Industry Research Division, East Sea Fisheries Research Institute, National Institute of Fisheries Science, Gangneung, South Korea; 3 Pathology Division, National Institute of Fisheries Science, Busan, South Korea; University of Southern California, Los Angeles, California, USA

**Keywords:** *Vibrio harveyi*, aquaculture, antibiotic resistance, whole-genome sequence, nanopore, olive flounder

## Abstract

*Vibrio harveyi* strain 22FBVib0145 was isolated from a diseased olive flounder farmed in Jeju, Korea. Here, we report the draft genome sequence of this strain. It is 6,238,277 bp in length with a G + C content of 44.8%.

## ANNOUNCEMENT

In April 2021, a diseased olive flounder (292 g, 30 cm) with hemorrhagic skin ulcer was collected from a fish farm in Seogwipo-si, Jeju-do, South Korea (33°16′16″N, 126°40′37″E). A *Vibrio harveyi* 22FBVib0145 was subsequently isolated by smearing the cross-section of the spleen onto tryptic soy (TS) agar (Oxoid, UK) and culturing for 24 hours at 28°C. In a minimum inhibitory concentration (MIC) test according to Clinical and Laboratory Standards Institute guideline VET04-A2 (1), 22FBVib0145 has been classified as non-wild type for amoxicillin (MIC >16 mg/L), oxytetracycline (MIC 64 mg/L), florfenicol (MIC 8 mg/L), and trimethoprim/sulfamethoxazole (MIC 2 mg/L/38 mg/L) based on epidemiological cut-off values proposed by Smith et al. (2). This isolate was stored at −80°C in TS broth containing 1% NaCl and 15% glycerol. Whole-genome sequencing was performed to characterize the genetic determinants for its multi-drug resistance profile.

A single colony of 22FBVib0145 from TS agar containing 1% NaCl was inoculated into TS broth containing 1% NaCl and incubated at 28°C for 18 hours. Genomic DNA was extracted from 1 mL of the culture for both short-read and long-read sequencing using a DNeasy blood and tissue kit (Qiagen, Germany). The gDNA was quantified using a Qubit v.3.0 fluorometer (Invitrogen, USA) and a Qubit dsDNA HS kit (Invitrogen) following the manufacturer’s protocol. A short-read sequencing library was prepared using a Nextera DNA Flex library preparation kit (Illumina, USA) and Nextera DNA CD indexes (Illumina) following the manufacturer’s protocol. A total of 1,260,804 paired-end reads (coverage ×27.6) were generated using an iSeq 100 instrument (Illumina) on paired-end sequencing (2 × 150 bp). A long-read sequencing library was prepared with a one-dimensional ligation sequencing kit SQK-LSK109 (Oxford Nanopore Technologies [ONT]) and a NEBNext Companion Module for ONT Ligation Sequencing kit (New England BioLabs, USA) following each manufacturer’s protocol. No size selection or shearing was applied. The library was loaded into a FLO-MIN106 flow cell (R9.4.1, ONT) on a MinION sequencer (ONT). Fast5 reads were base then base-called using a MinKNOW v.19.10.1 (ONT) with Guppy v.3.3.3 in high-accuracy base-calling mode, generating 1,824,000 reads (coverage ×269.3) with an *N*_50_ of 1,919 bp. FastQC v.0.11.7 (3) was employed to assess the sequence quality of both Illumina and ONT libraries. Hybrid *de novo* assembly was performed using Unicycler v.0.4.8 (4). The assembly quality was evaluated with QUAST v.5.0.2 (5). Genome annotations were carried out with National Center for Biotechnology Information Prokaryotic Genome Annotation Pipeline v.6.3 (6). Default parameters were used for all software unless otherwise specified.

The 22FBVib0145 genome was 6,158,475 bp in length, with a G + C content of 45.09%. It includes 2,989 predicted protein-coding regions, 129 tRNA genes, and 37 rRNAs distributed in seven contigs (3,586,044 bp, *N*_50_ value) containing two circular chromosomes (3,586,044 and 2,290,217 bp, respectively) and five linear contigs. As a result of average nucleotide identity (ANI) analysis using the JSpecies v.4.0.2 (7), the sequence showed a 98.76% ANI with the *V. harveyi* American Type Culture Collection 33843, a type strain. A BLASTx search using Resistance Gene Identifier v.6.0.2 for perfect, strict, and loose hits within the Comprehensive Antibiotic Resistance Database v.3.2.8 (8) showed that 22FBVib0145 carried genes associated with antibiotic resistance (amino acid identity), including VHH-1 (91.52%), *tet*(B) (99.75%), *tet*(M) (95.46%), *floR* (99.5%), and *sul2* (100%).

## Data Availability

Raw sequences of both Illumina and Nanopore reads were submitted to the National Center for Biotechnology Information Sequence Read Archive with accession numbers SRR21885129 and SRR21885128 under BioProject number PRJNA889991 and BioSample number SAMN31264238. The assembled genome sequence of 22FBVib0145 was deposited in GenBank with accession number JAOWIO000000000.1.
